# Supplementary material for: Exciton engineering of 2D Ruddlesden–Popper perovskites by synergistically tuning the intra and interlayer structures
Source: Nat Commun. 2024 Apr 8;15:3001. doi: 10.1038/s41467-024-47225-4 (PMC11001939; doi:10.1038/s41467-024-47225-4)

Supplementary Information

Exciton engineering of 2D Ruddlesden–Popper perovskites by synergistically tuning the intra and interlayer structures

Songhao Guo,[1] Willa Mihalyi-Koch,[2] Yuhong Mao,[1] Xinyu Li,[3] Kejun Bu,[1] Huilong Hong,[3] Matthew P. Hautzinger,[2] Hui Luo,[1] Dong Wang,[1] Jiazhen Gu,[3] Yifan Zhang,[1] Dongzhou Zhang,[4] Qingyang Hu,[1] Yang Ding,[1] Wenge Yang,[1] Yongping Fu,[3,] Song Jin,[2,*] and Xujie Lü[1,*]*

[1] Center for High Pressure Science and Technology Advanced Research (HPSTAR), Shanghai, China

[2] Department of Chemistry, University of Wisconsin-Madison, Madison, Wisconsin, United States

[3] Beijing National Laboratory for Molecular Science, College of Chemistry and Molecular Engineering, Peking University, Beijing, China

[4] Hawaii Institute of Geophysics & Planetology, University of Hawaii Manoa, Honolulu, Hawaii, United States

Supplementary Discussion

Determination of the photo-generated carrier density.

The photo-generated carrier density (N_0) of the samples can be evaluated by following equation:

$$N_0 = \frac{E\lambda\alpha(\lambda)}{hcA_{\mathrm{eff.}}} \times \left(1 - R_{\mathrm{pump}}\right) \times \eta_{\mathrm{d}}(\lambda) \tag{1}$$

where E is the energy of the excited laser. λ, $\alpha(\lambda)$ and R_{pump} represents the wavelength, absorption coefficient, and reflectivity of the excited laser, respectively. $A_{\mathrm{eff.}}$ is the light spot effective area. η_{d} is the transmittance of the diamond.

The value of absorption coefficient $\alpha(\lambda)$ can be obtained from UV-Vis measurements and calculated as follows:

$$\alpha(\lambda) = -\frac{1}{l}\ln\frac{T}{1-R} \tag{2}$$

where T, R and l is the transmittance, reflectivity, and thickness of the perovskite thin flake, respectively.

Quantification of exciton-phonon coupling.

The photoluminescence (PL) line width at each pressure were fitted according to the following equation:[1]

$$\Gamma(T) = \Gamma_0 + \frac{\gamma_{\mathrm{LO}}}{e^{\frac{E_{\mathrm{LO}}}{kT}} - 1} \tag{3}$$

The first term, Γ_0, is the inhomogeneous broadening term and the line width at 0 K. The second term describes the homogeneous broadening due to the longitudinal optical (LO) phonon scattering *via* Fröhlich interaction, where γ_{LO} is coupling strength and E_{LO} represents the phonon energy.

Calculation of photoconductivity.

The photo-conductivities were calculated using the following equation:

$$\sigma = \frac{I \times l}{U \times S} \tag{4}$$

where U is bias voltage, I is photocurrent, and S and l are the cross-sectional area and distance of the sample between the two electrodes, respectively.

Calculation of effective masses.

The effective masses of the electron and hole were derived from the following expression:

$$m^* = \hbar^2 \left[\frac{\partial^2 \varepsilon(k)}{\partial k^2}\right]^{-1} \tag{5}$$

where the k is the wave vector along the transport direction, $\varepsilon(k)$ represents the energy band eigenvalues, and the $\hbar$ is the reduced Planck constant.

Calculation of exciton binding energy.

According to the Wannier exciton model,[2] the exciton binding energy (E_b) is given as the following forms:

$$E_\mathrm{b} = \frac{e^4}{2(4\pi\varepsilon_0)^2\hbar^2} \times \frac{m_\mathrm{r}^*}{\varepsilon_\infty^2} \tag{6}$$

where m_r^* is the exciton reduced mass based on the equation,

$$\frac{1}{m_\mathrm{r}^*} = \frac{1}{m_\mathrm{e}} + \frac{1}{m_\mathrm{h}} \tag{7}$$

and ε_∞ is the static dielectric constant contributed from the electron.

Calculation of perovskite cage volume, layer distance, and structural descriptor χ.

The area of the bottom square facet of the perovskite cage in a 2D RP perovskite is calculated

according to the following equation:

$$S = a \times b \times \sin\theta \tag{8}$$

where *a* and *b* are the equatorial Pb-Pb distances along two directions, *θ* is the Pb-Pb-Pb angle.

Then, the cage volume *V* is calculated according to the following equation:

$$V = S \times c \times \sin\gamma \tag{9}$$

where *c* is the axial Pb-Pb distance and *γ* is the angle between axial Pb-Pb and the bottom square facet of the perovskite cage. The cage volume *V* in *n* = 3 and *n* = 4 2D perovskites are the average value among different cages.

Layer distance *L* is defined as the interlayer distance between Pb atoms in adjacent layers.

Consequently, the structural descriptor χ can be determined using the following equation:

$$\chi = V \times \frac{L}{\sqrt{N}} \tag{10}$$

where *N* is the number of non-hydrogen atoms in the interlayer spacer cations. The *N* values of BA, HA, and CMA are 5, 7, and 8, respectively.

Correlation analysis

The correlation between PL symmetric factor and various structural parameters can be estimated by correlation coefficient. The correlation coefficient $\rho_{X,Y}$ between two random variables *X* and *Y* with expected values $\bar{X}$ and $\bar{Y}$ and standard deviations σ_X and σ_Y is defined as:

$$\rho_{\mathrm{X,Y}} = \frac{cov(X,Y)}{\sigma_{\mathrm{X}}\sigma_{\mathrm{Y}}} = \frac{E[(X-\bar{X})(Y-\bar{Y})]}{\sigma_{\mathrm{X}}\sigma_{\mathrm{Y}}} \tag{11}$$

where *E* is the expected value operator, *cov* means covariance. The value of a correlation coefficient ranges between −1 and +1. The correlation coefficient is +1 in the case of a perfect

direct (increasing) linear relationship (correlation), −1 in the case of a perfect inverse (decreasing) linear relationship (anti-correlation). As it approaches zero there is less of a relationship (closer to uncorrelated). The closer the coefficient is to either −1 or 1, the stronger the correlation between the variables.

Firstly, in terms of intralayer structure, compression induces a decrease in the Pb-I bond length and an increase in the Pb-I-Pb bond angle. We plot the PL symmetric factor as a function of average Pb-I bond length, only getting a correlation coefficient $\rho = -0.789$ (Supplementary Figure 14a). Compression also leads to an increase in the Pb-I-Pb bond angle, thus resulting in an idealization of the perovskite cage and a subsequent reduction in its cage volume (V). The correlation coefficient improves to −0.904 when we use cage volume as the structural descriptor (Supplementary Figure 14b).

Then, in terms of interlayer structure, compression induces a decrease in the interlayer distances. However, no clear correlation can be found between PL symmetric factor and interlayer distances (Supplementary Figure 14c). The probable reason is because interlayer distance only depicts the crystal structure and do not consider the dielectric constant of the spacer cations. For example, HA and BA fall on different trendlines because HA has two extra C atoms and causes a larger interlayer distance, but their dielectric constants are very similar. Therefore, we choose packing density of the spacer cations instead of simple interlayer distance, which essentially convey how well the spacer cations pack and are normalized from interlayer distance as follows:

$$\text{Packing density} = L \times N^{\alpha} \tag{12}$$

where the L and N are interlayer distance and number of non-hydrogen atoms in the spacer cation, respectively. The correlation coefficient between PL symmetric factor and packing density as a function of exponential value α is shown in Supplementary Figure 15, where the strongest correlation can be found when the α near −0.5. Thus, the packing density in this case can be defined

as $\frac{L}{\sqrt{N}}$, which shows a much better correlation coefficient $\rho = -0.686$ with PL symmetric factor (Supplementary Figure 14d).

A further improved correlation coefficient can be achieved when we synergistically considered both the intra- and interlayer structural parameters, obtaining a new comprehensive structural descriptor χ:

$$\chi = \text{Cage volume} \times \text{Packing density} = V \times \frac{L}{\sqrt{N}} \tag{13}$$

where V and L refer to the perovskite cage volume and interlayer distance, respectively, and N is the number of non-hydrogen atoms in the interlayer spacer cation. The correlation coefficient between PL symmetric factor and this structural descriptor χ reaches −0.961 (Supplementary Figure 14e), which indicates a very good correlation. The step-by-step correlation process above also confirms that each component of χ (V, L, and $\sqrt{N}$) is essential for achieving such a universal correlation.

Synergistic effects of intra and interlayer structures

The term synergistic refers to the interaction of two or more elements in a way that their combined effect is greater than the sum of their individual effects. To further illustrate the synergistic effects of intra- and interlayer structures, we step-by-step introduced a smaller MA and a shorter iBA to substitute the GA and BA cations in the intra and interlayer of $(BA)_2(GA)Pb_2I_7$, respectively, as shown in Supplementary Figure 20a. The synergistic effect is demonstrated in both structure tuning and property optimization.

From the aspect of structure, whether involving intra or interlayer cation-substitution, there is limited tuning of the structural descriptor χ, where only concurrent intra and interlayer cation-substitution achieves the optimized χ value (Supplementary Figure 20b). From the aspect of

property, as shown in Supplementary Figure 20c, individual intra (BA-MA) or interlayer (iBA-GA) cation-substitution leads to a 14.3- or 2.6-times PL increments, respectively, while 27.9-times increment (obviously larger than 14.3+2.6-1=15.9 times) is achieved through concurrent intra and interlayer cation-substitution (iBA-MA). Similar results have been obtained for other systems, as shown in Supplementary Figures 20 d and e.

Thus, from both the structural tuning and property optimization, it is revealed that the combined effect is more effective than the sum of individual intra or interlayer cation-substitution, according with the term synergistic.

Supplementary Tables

Supplementary Table 1. Crystallographic information for $(BA)_2(GA)Pb_2I_7$ at selected pressures.

Pressure	0.4 GPa	2.9 GPa	5.5 GPa
Crystal system	Monoclinic	Monoclinic	Monoclinic
Space group	*C*2/*c*	*C*2/*c*	*C*2/*c*
Cell parameters	a = 37.860 Å<br>b = 9.1394 Å,<br>c = 8.8393 Å<br>β = 93.41°	a = 35.385 Å<br>b = 8.8763 Å,<br>c = 8.4755 Å<br>β = 94.05°	a = 34.569 Å<br>b = 8.7362 Å,<br>c = 8.3596 Å<br>β = 93.88°
Volume	3053.1387 Å^3	2655.3742 Å^3	2518.8562 Å^3

Supplementary Table 2. Crystal data and structure refinement for $(CMA)_2(FA)Pb_2I_7$.

Empirical formula	$C_{15}H_{37}I_7N_4Pb_2$
Formula weight	1576.16
Temperature/K	295.00
Crystal system	orthorhombic
Space group	*Pnma*
a/Å	8.8914(11)
b/Å	44.831(6)
c/Å	8.8855(9)
α/°	90
β/°	90
γ/°	90
Volume/Å^3	3541.8(7)
Z	4
ρ_{calc} g/cm^3	2.956
μ/mm^{-1}	66.244
F(000)	2760.0
Crystal size/mm^3	0.04 × 0.04 × 0.01
Radiation	Cu Kα (λ = 1.54178)
2Θ range for data collection/°	3.942 to 158.302
Index ranges	-11 ≤ h ≤ 9, -55 ≤ k ≤ 46, -10 ≤ l ≤ 11
Reflections collected	22774
Independent reflections	4048 [R_{int} = 0.0920, R_{sigma} = 0.0635]
Data/restraints/parameters	4048/102/142
Goodness-of-fit on F^2	1.042
Final R indexes [I>=2σ (I)]	R_1 = 0.0586, wR_2 = 0.1532
Final R indexes [all data]	R_1 = 0.0754, wR_2 = 0.1640
Largest diff. peak/hole / e Å^{-3}	2.55/-2.92

Supplementary Figures

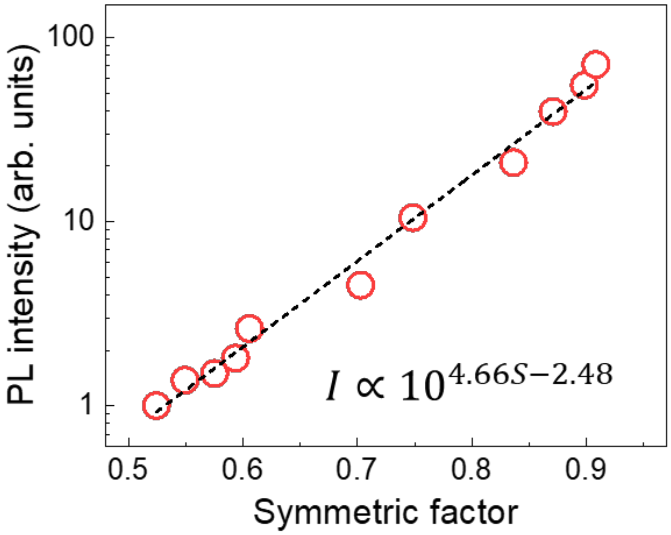

Supplementary Fig. 1 Analysis of PL spectra. The relative PL intensity of $(BA)_2(GA)Pb_2I_7$ as a function of the PL symmetric factor.

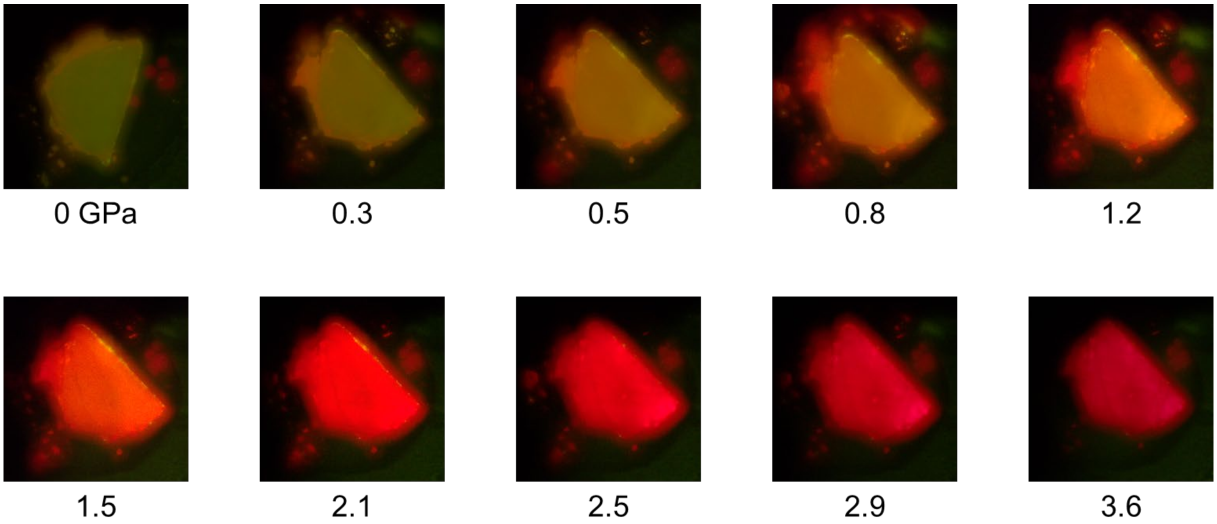

Supplementary Fig. 2 Fluorescence micrographs. Fluorescence micrographs of a $(BA)_2(GA)Pb_2I_7$ single crystal flake upon compression, where the gradually enhanced emission up to 2.1 GPa can be observed visually. The total field of view of micrograph is 100 μm.

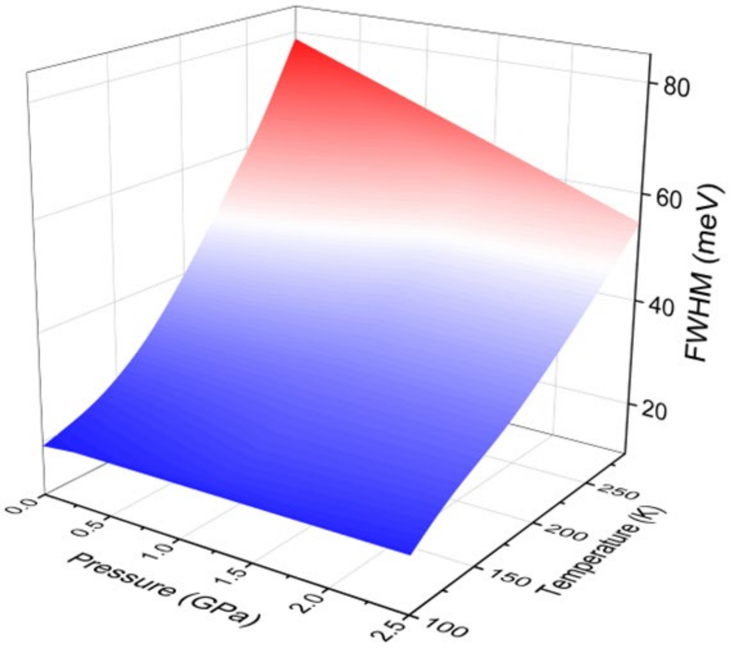

Supplementary Fig. 3 Temperature- and pressure-dependent PL line width. Dependence of the PL line width (full width half max, FWHM) of $(BA)_2(GA)Pb_2I_7$ on temperature and pressure in a 2D color plot, indicating that the thermal broadening of PL line width is suppressed upon compression.

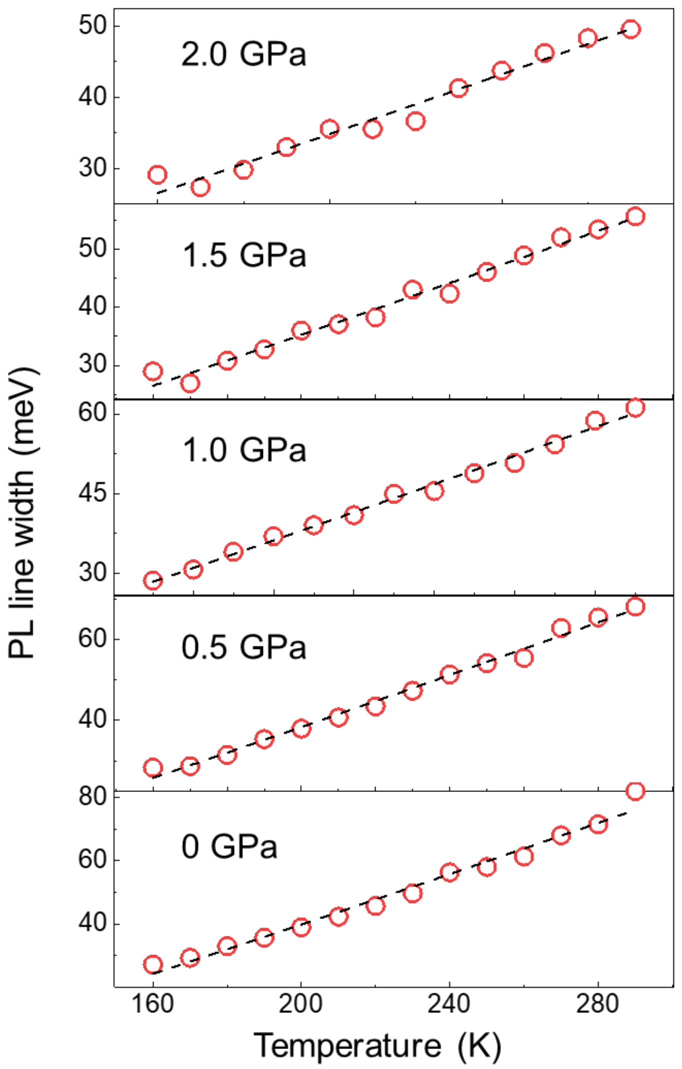

Supplementary Fig. 4 Fitting results of Temperature-dependent PL line width. PL line width as a function of temperature of $(BA)_2(GA)Pb_2I_7$ at different pressures. The fitting curves according to Equation 3 are shown in dash lines.

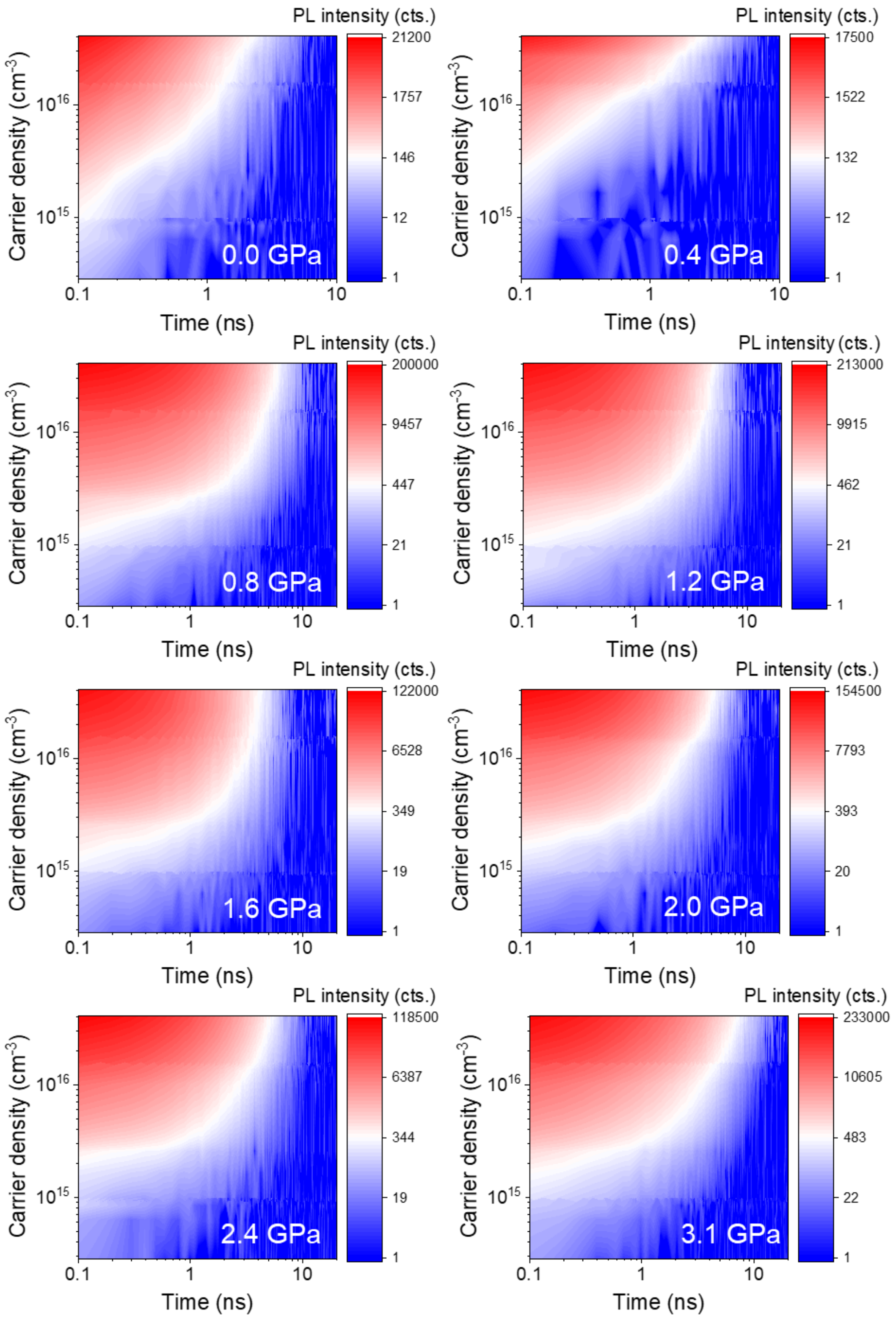

Supplementary Fig. 5 Time-resolved PL spectra. Time resolved PL spectra as a function of carrier density N_0 for $(BA)_2(GA)Pb_2I_7$ at different pressures.

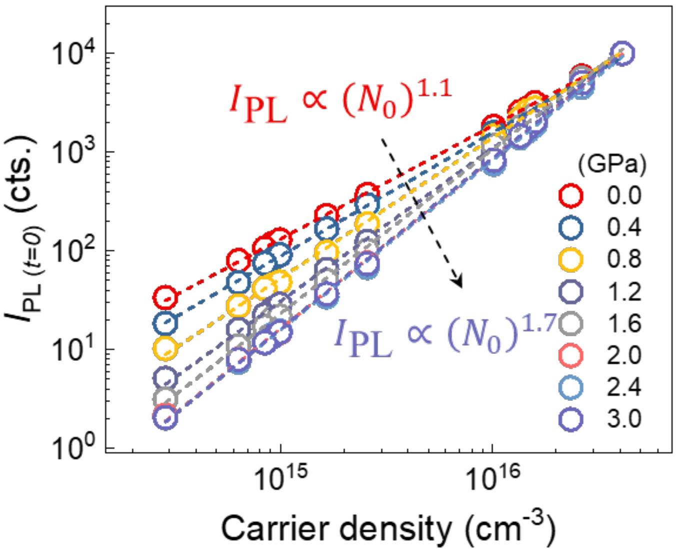

Supplementary Fig. 6 Fitting results of time-resolved PL spectra. The initial time PL intensity versus photon-injected carrier density of $(BA)_2(GA)Pb_2I_7$ as a function of pressure. The plots follow the relationship $I_{\mathrm{PL}}(t = 0) \propto (N_0)^{\alpha}$ (dash lines), where the fitted exponential value α indicates the major carrier specie ($\alpha = 1$ implies excitons, $\alpha = 2$ implies free carriers, and α between 1 and 2 suggests the coexisting of excitons and free carriers).

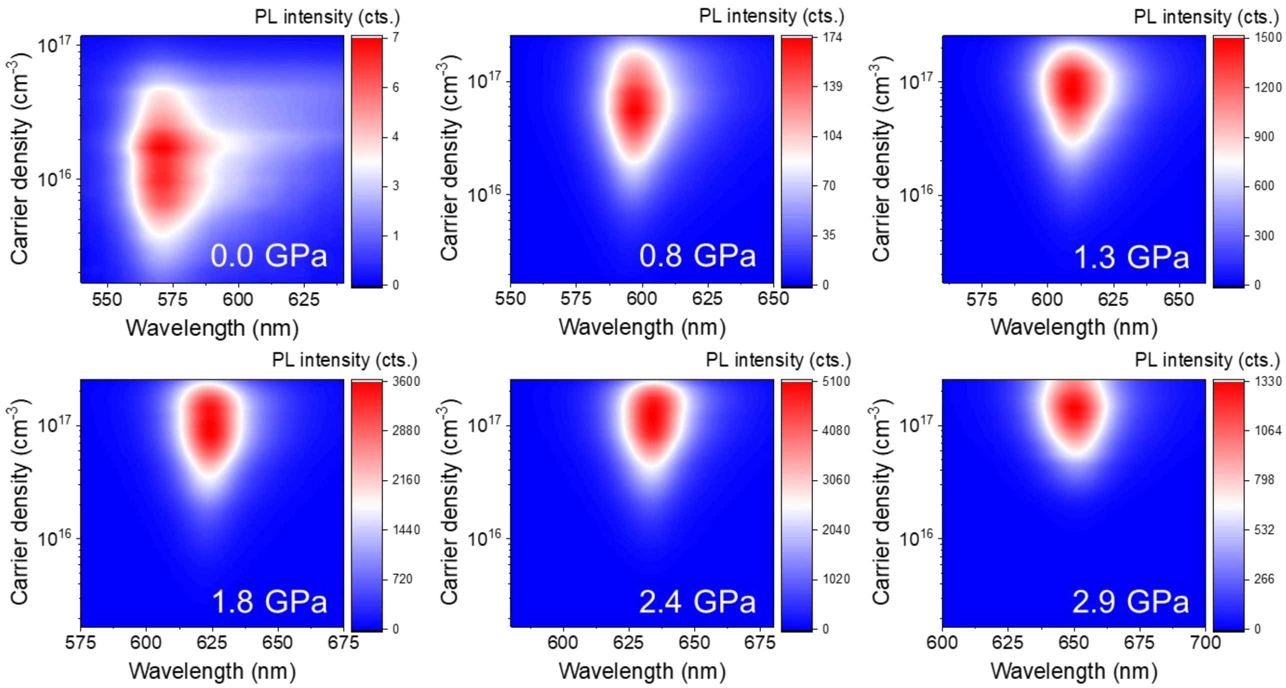

Supplementary Fig. 7 Power-dependent PL spectra. Power-dependent PL spectra of $(BA)_2(GA)Pb_2I_7$ under different pressures.

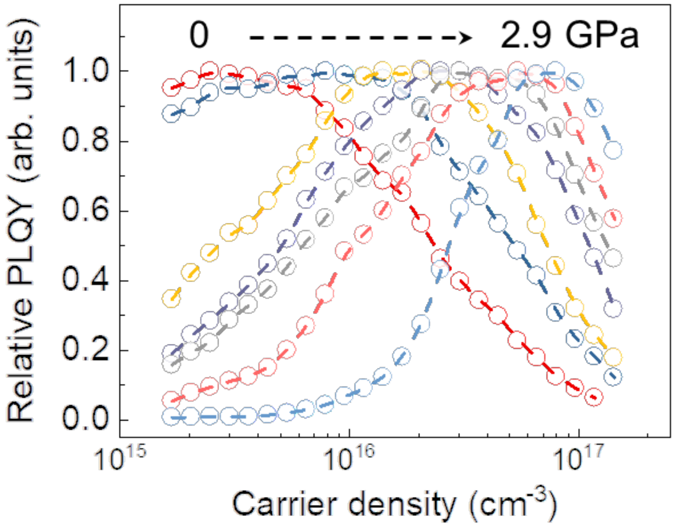

Supplementary Fig. 8 PL quantum yield. Relative PL quantum yield (PLQY) for $(BA)_2(GA)Pb_2I_7$ as a function of carrier density at different pressures.

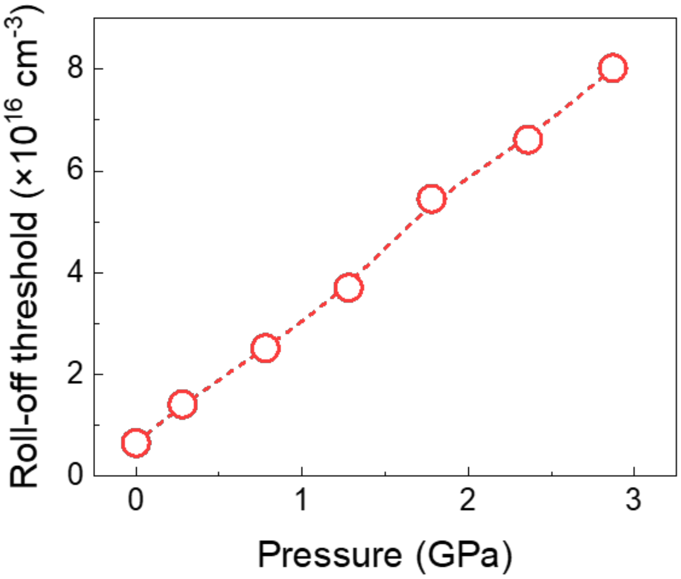

Supplementary Fig. 9 Efficiency roll-off threshold. The efficiency roll-off threshold of $(BA)_2(GA)Pb_2I_7$ as a function of pressure, which is substantially increased by more than one order of magnitude upon compression. This observation indicates a suppressed Auger recombination due to the weakening of Coulombic interaction.

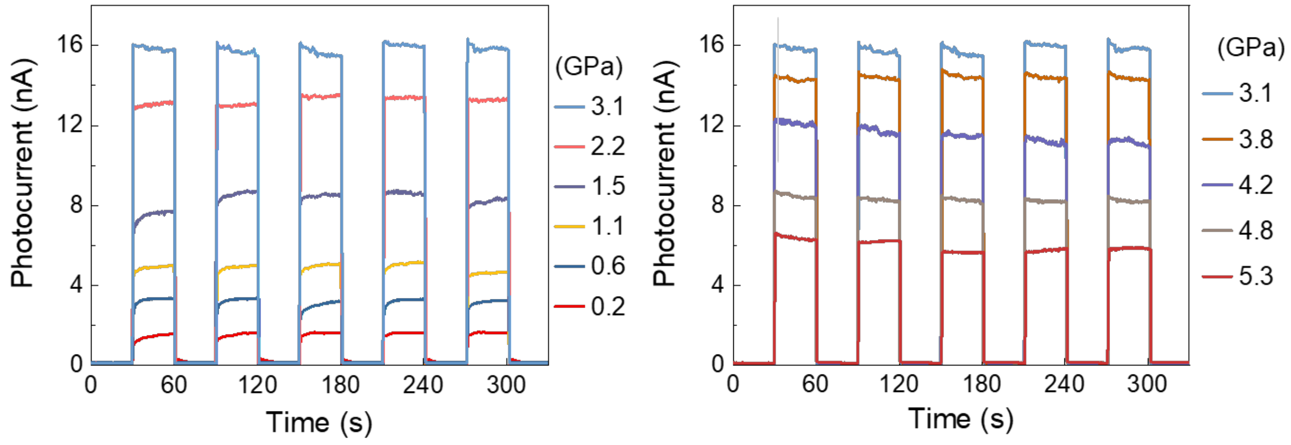

Supplementary Fig. 10 Photo-response. Photocurrents of $(BA)_2(GA)Pb_2I_7$ under high pressure, which exhibit fast on-off switch responses to light illumination. The photocurrent gradually increases with pressure up to ~3 GPa.

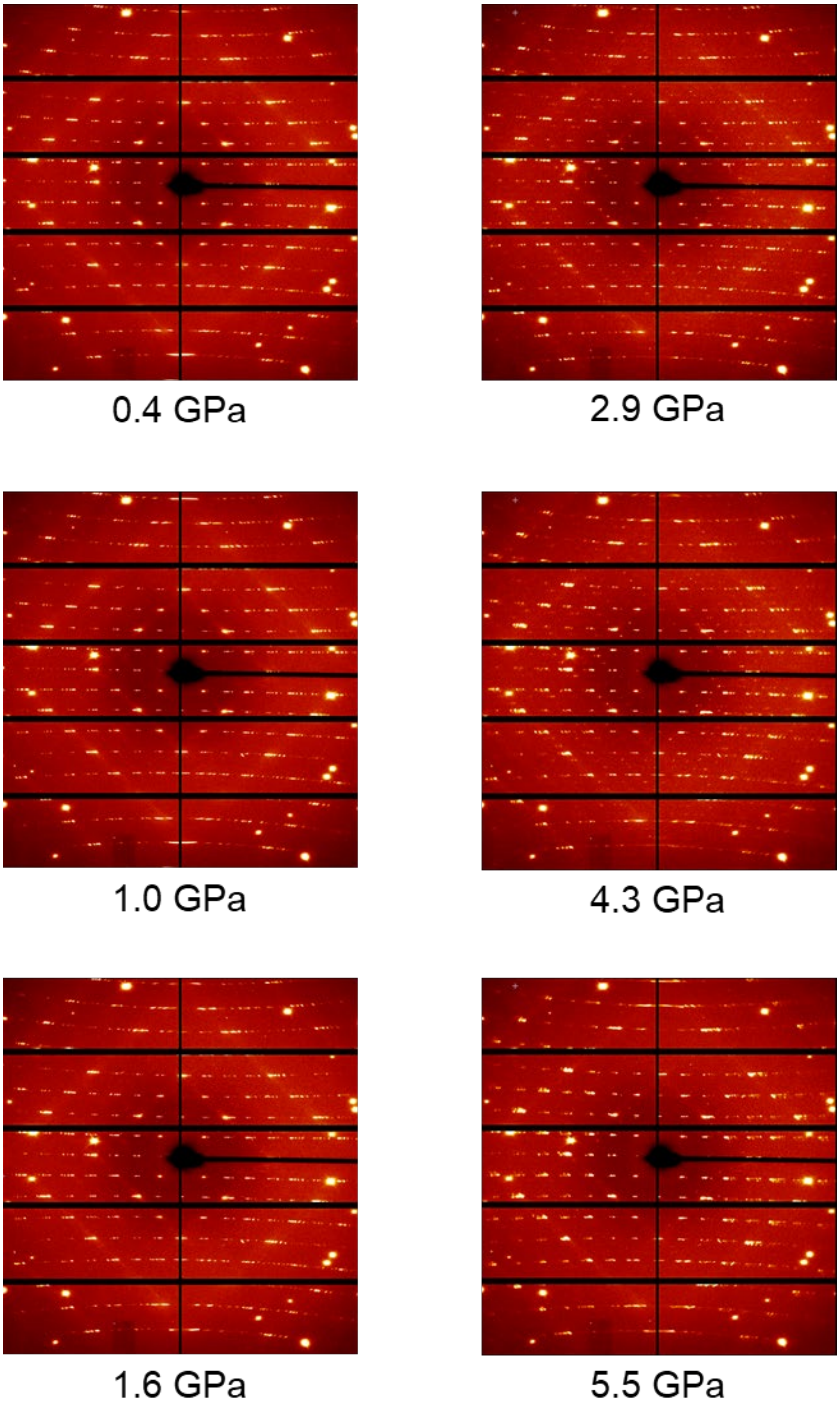

Supplementary Fig. 11 Single-crystal X-ray diffraction. Single-crystal X-ray diffraction images of $(BA)_2(GA)Pb_2I_7$ at different pressures.

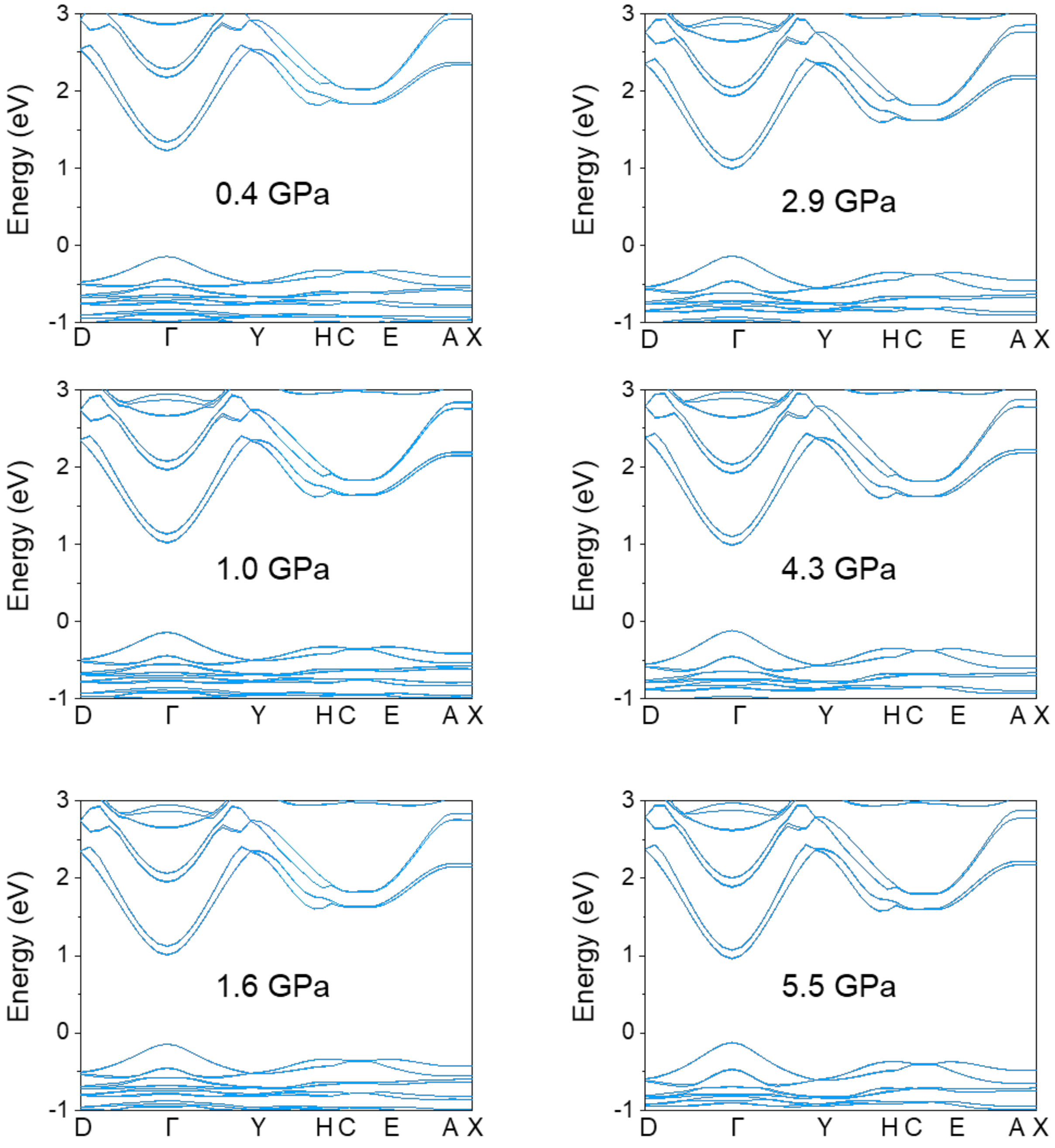

Supplementary Fig. 12 Calculated electronic structure. Calculated electronic structures of $(BA)_2(GA)Pb_2I_7$ at different pressures, which reveal a more dispersive nature near the band edges and suggest an enhanced carrier mobility at a higher pressure. The band structures at 0.4 and 2.9 GPa are also shown in Figure 3d of the main text.

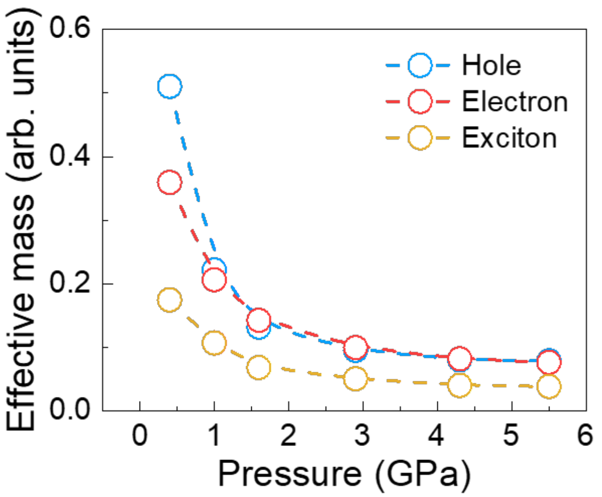

Supplementary Fig. 13 Effective mass. The calculated effective masses of electron and hole as well as the exciton reduced mass of $(BA)_2(GA)Pb_2I_7$ as a function of pressure. The exciton reduced mass was calculated according to Equation 7.

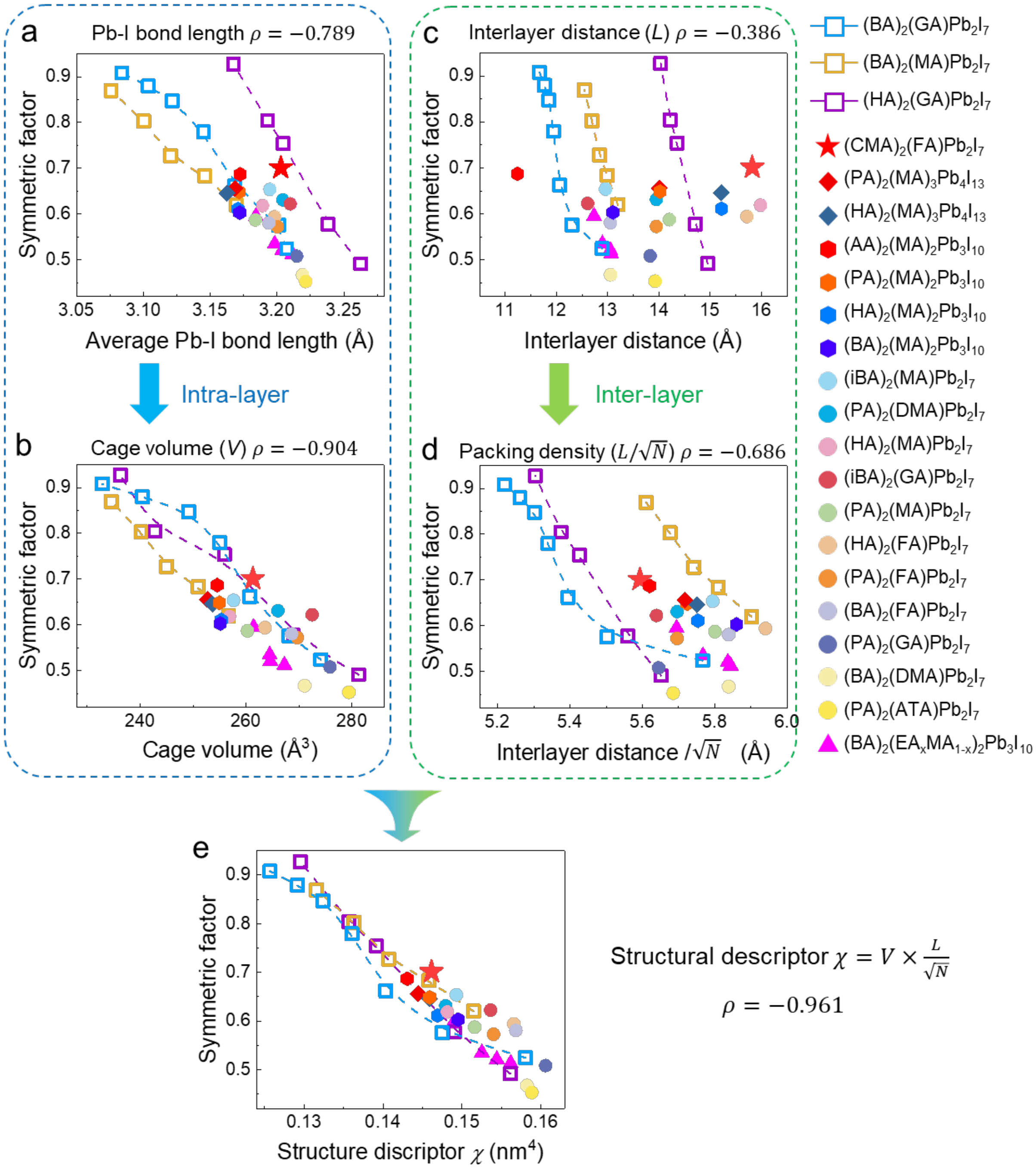

Supplementary Fig. 14 Structural descriptor. PL symmetric factor as a function of (**a**) average Pb-I bond length, (**b**) cage volume V, (**c**) interlayer distance, (**d**) packing density, and (**e**) structural descriptor χ for various $n \geq 2$ 2D RP perovskites (listed in the legends). The correlation coefficients are given for each plot. Comprehensive consideration of both the intralayer (V) and interlayer (L and $\sqrt{N}$) structural characteristics in the proposed structural descriptor χ is essential for achieving a good correlation.

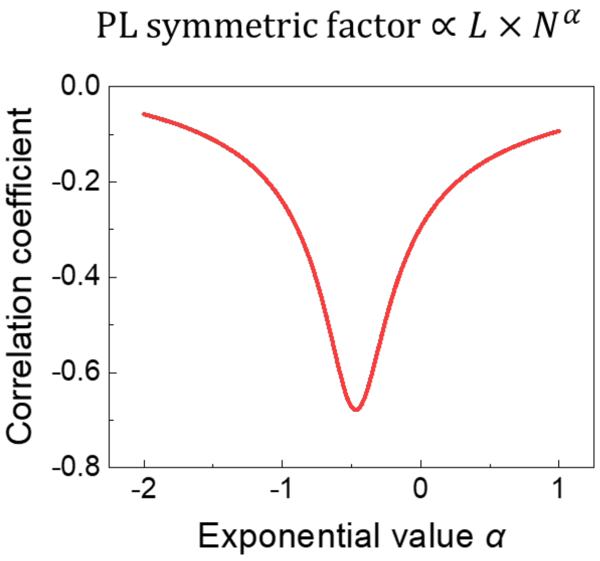

Supplementary Fig. 15 Correlation analysis. The correlation coefficient between PL symmetric factor and packing density as a function of the exponential value α of N, where the strongest correlation can be found when the α near −0.5.

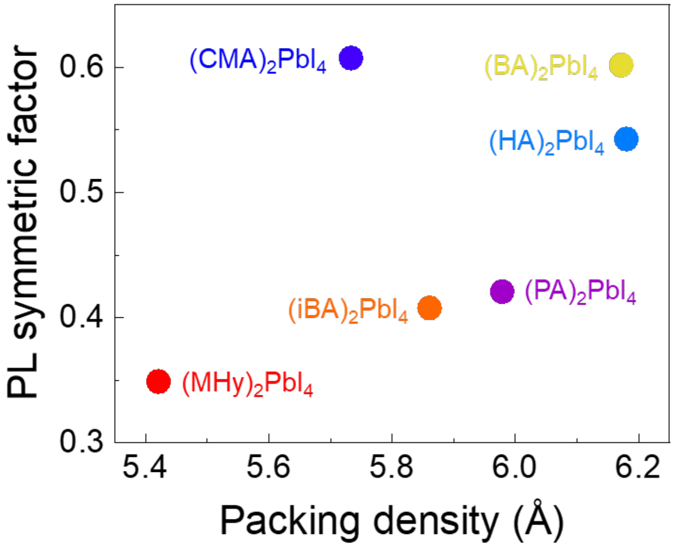

Supplementary Fig. 16 Structural-property relationship for *n* = 1 2D perovskites. The PL symmetric factor as a function of packing density $\frac{L}{\sqrt{N}}$ for various $n = 1$ 2D perovskites, where no clear correlation can be found.

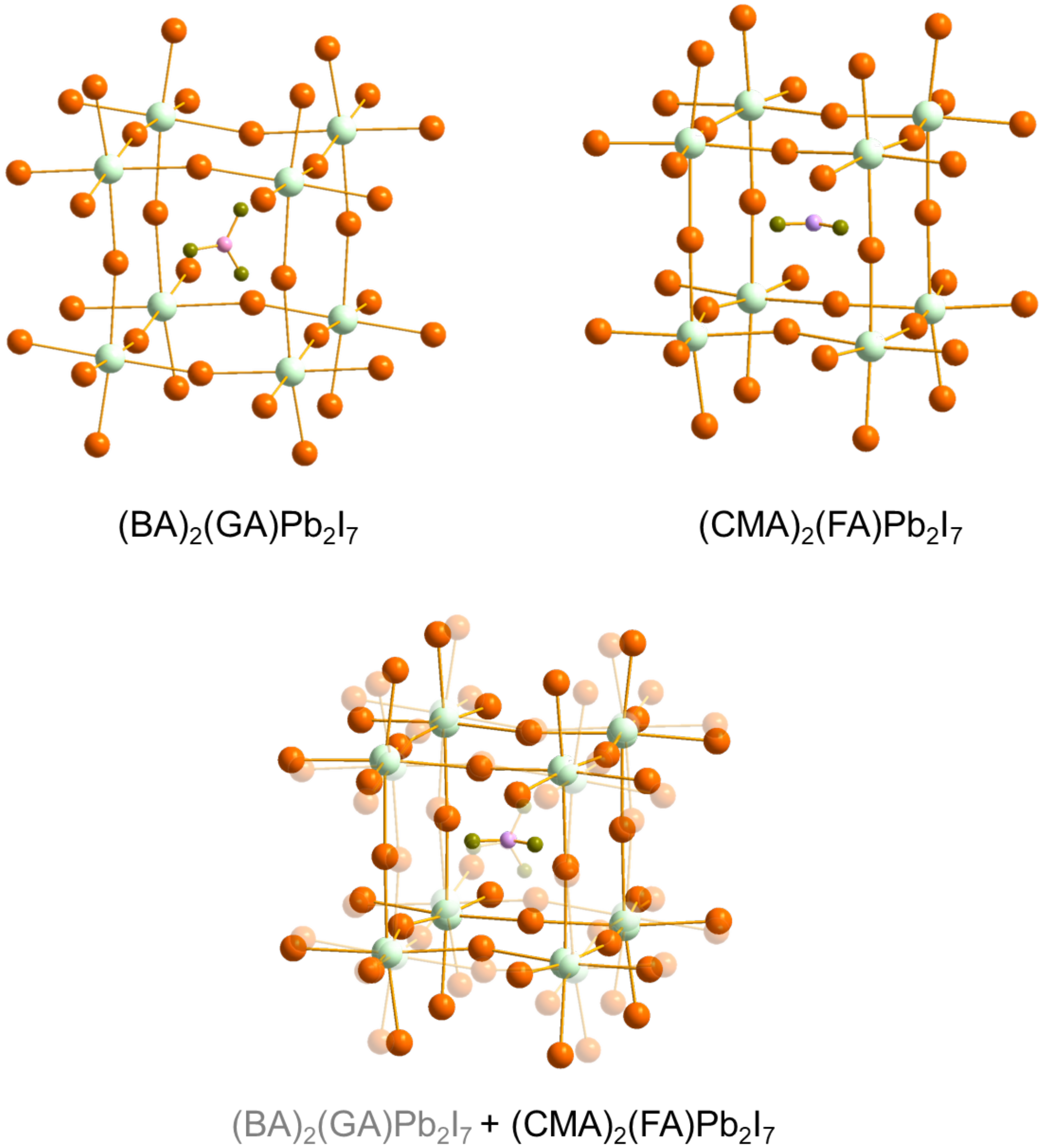

Supplementary Fig. 17 Crystal structure. Crystal structures of $(BA)_2(GA)Pb_2I_7$ and $(CMA)_2(FA)Pb_2I_7$ at ambient conditions, which show the occupation of the GA and FA cation in the perovskite cage formed by eight $[PbI_6]^{4-}$ octahedra. The overlapped crystal structures highlight the differences between the two structures.

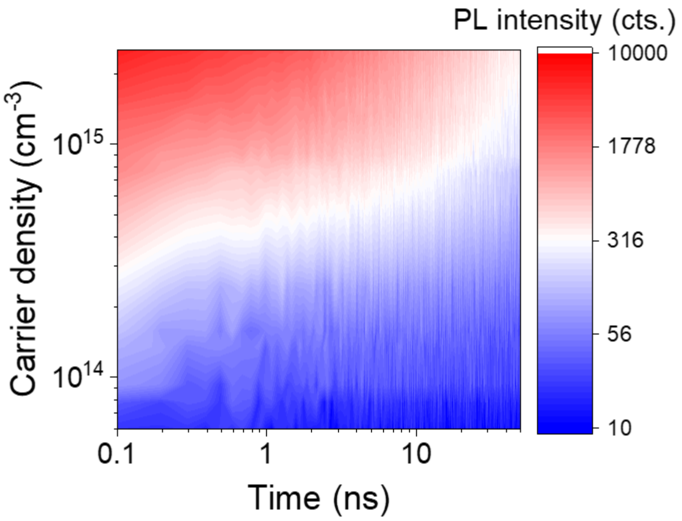

Supplementary Fig. 18 Time-resolved PL spectra. Time-resolved PL spectra as a function of carrier density N_0 for $(CMA)_2(FA)Pb_2I_7$.

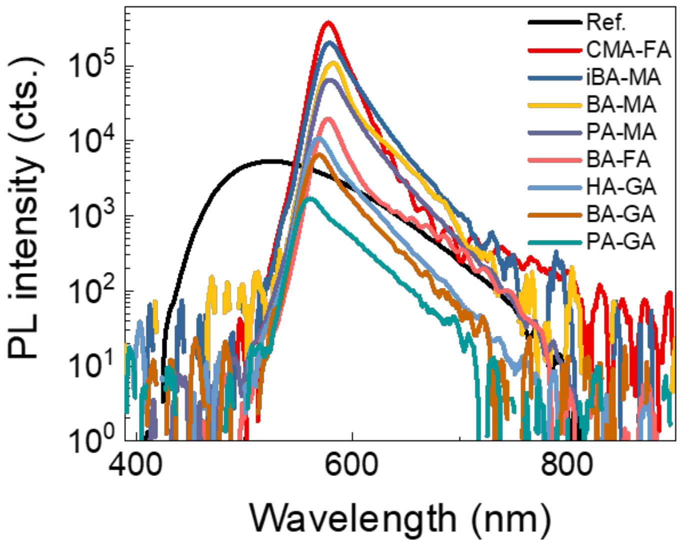

Supplementary Fig. 19 PLQY measurement. PL intensity for various common n = 2 RP perovskites $(LA)_2APb_2I_7$ (LA-A, LA = CMA, HA, PA, BA, and iBA, A = GA, FA, and MA) and the reference sample Alq_3. The PLQY can be estimated according to Equation 1 in the main text and the PLQY values were plotted in Figure 5d.

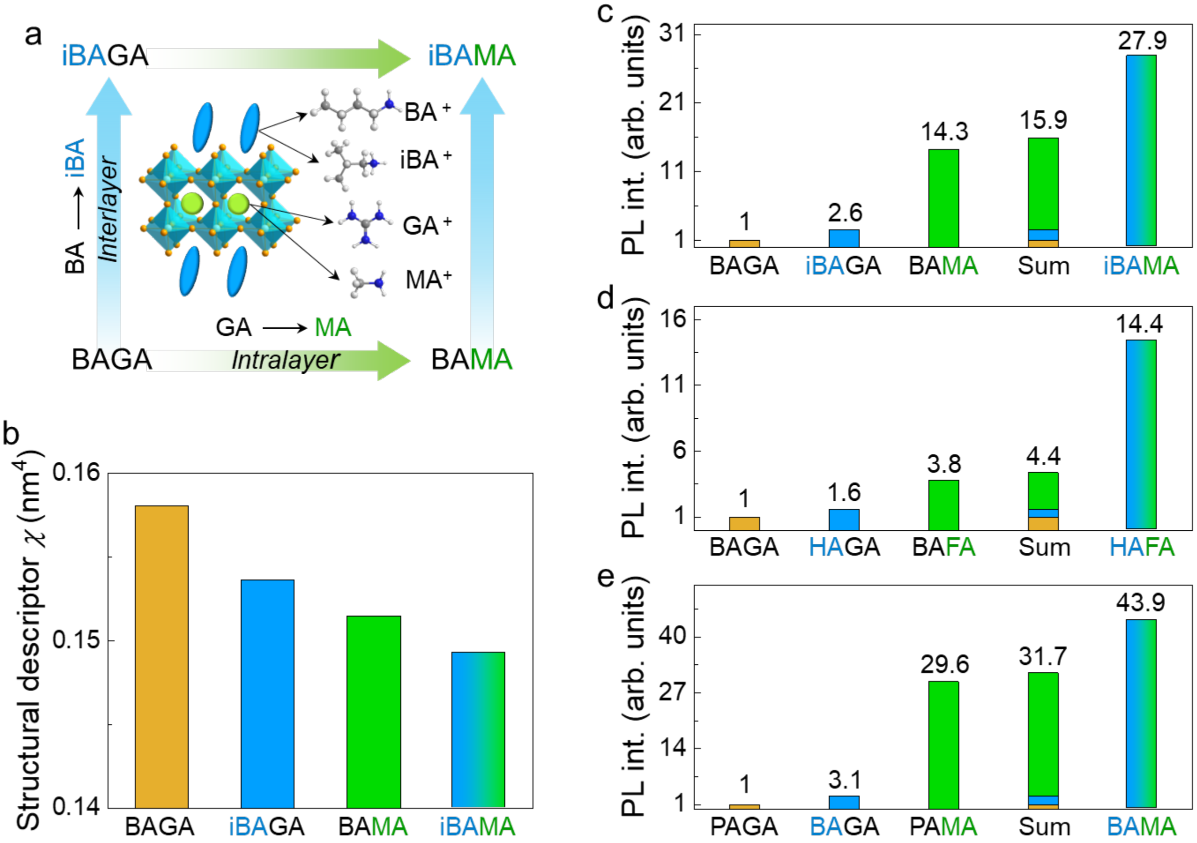

Supplementary Fig. 20 Synergistic manipulation of inter and intralayer structures. (**a**) Schematic illustration of intra and interlayer cation substitutions to achieve a synergistic structure and property tuning. (**b**) The values of structural descriptor χ for the various cation-substituted 2D perovskites, where concurrent intra and interlayer cation-substitution achieves the optimal χ value. (**c-e**) Relative PL intensity for various 2D perovskite systems with step-by-step cation substitutions. The PL intensity for concurrent intra and interlayer cation-substitution is larger than the sum of the PL intensities for individual intra or interlayer cation-substitution, further illustrating the synergistic effect.

Supplementary References

1 Wright, A. D. *et al.* Electron-phonon coupling in hybrid lead halide perovskites. *Nat. Commun.* **7**, (2016).

2 Jong, U.-G., Yu, C.-J., Ri, J.-S., Kim, N.-H. & Ri, G.-C. Influence of halide composition on the structural, electronic, and optical properties of mixed CH3NH3Pb(I1-xBrx)3 perovskites calculated using the virtual crystal approximation method. *Phys. Rev. B* **94**, 125139, (2016).

checkCIF/PLATON report

Structure factors have been supplied for datablock(s) jin165_d

THIS REPORT IS FOR GUIDANCE ONLY. IF USED AS PART OF A REVIEW PROCEDURE FOR PUBLICATION, IT SHOULD NOT REPLACE THE EXPERTISE OF AN EXPERIENCED CRYSTALLOGRAPHIC REFEREE.

No syntax errors found. CIF dictionary Interpreting this report

Datablock: jin165_d

Bond precision:	C-C = 0.0371 A	Wavelength=1.54178	
Cell:	a=8.8914(11)	b=44.831(6)	c=8.8855(9)
	alpha=90	beta=90	gamma=90
Temperature:	295 K		

	Calculated	Reported
Volume	3541.9(7)	3541.8(7)
Space group	P n m a	P n m a
Hall group	-P 2ac 2n	-P 2ac 2n
Moiety formula	I7 Pb2, 2(C7 H16 N), C H5 N2	I7 Pb2, 2(C7 H16 N), 2(C0.5 H2.5 N)
Sum formula	C15 H37 I7 N4 Pb2	C15 H37 I7 N4 Pb2
Mr	1576.19	1576.16
Dx,g cm-3	2.956	2.956
Z	4	4
Mu (mm-1)	66.243	66.244
F000	2760.0	2760.0
F000’	2731.76	
h,k,lmax	11,57,11	11,55,11
Nref	3891	4048
Tmin,Tmax	0.134,0.516	0.039,0.178
Tmin’	0.037	

Correction method= # Reported T Limits: Tmin=0.039 Tmax=0.178
AbsCorr = MULTI-SCAN

Data completeness= 1.040 Theta(max)= 79.151

R(reflections)= 0.0585(3128) wR2(reflections)= 0.1639(4048)

S = 1.042 Npar= 142

The following ALERTS were generated. Each ALERT has the format
test-name_ALERT_alert-type_alert-level.
Click on the hyperlinks for more details of the test.

Alert level B

PLAT342_ALERT_3_B Low Bond Precision on C-C Bonds .... 0.03714 Ang.

Author Response: The E-map is dominated by heavy atoms and so the peak positions for the light atoms are not as well defined. The data quality is high and this was the best achievable precision with the data acquired.

Alert level C

PLAT042_ALERT_1_C Calc. and Reported MoietyFormula Strings Differ Please Check

Author Response: The calc. and reported moiety formula strings are equivalent.

PLAT241_ALERT_2_C High 'MainMol' Ueq as Compared to Neighbors of C1 Check

Author Response: The dataset was collected at 295 K, therefore there is more vibrational motion of the atoms leading to larger displacement ellipsoids. This is especially the case for atoms with more vibrational/rotational freedom (such as N1, C1) or for atoms in disordered molecules (N2, N3). However, modeling these atoms as disordered did not improve the model

PLAT241_ALERT_2_C High 'MainMol' Ueq as Compared to Neighbors of C5 Check

Author Response: The dataset was collected at 295 K, therefore there is more vibrational motion of the atoms leading to larger displacement ellipsoids. This is especially the case for atoms with more vibrational/rotational freedom (such as N1, C1) or for atoms in disordered molecules (N2, N3). However, modeling these atoms as disordered did not improve the model

PLAT241_ALERT_2_C High 'MainMol' Ueq as Compared to Neighbors of N2 Check

Author Response: The dataset was collected at 295 K, therefore there is more vibrational motion of the atoms leading to larger displacement ellipsoids. This is especially the case for atoms with more vibrational/rotational freedom (such as N1, C1) or for atoms in disordered molecules (N2, N3). However, modeling these atoms as disordered did not improve the model

```
PLAT241_ALERT_2_C High   'MainMol' Ueq as Compared to Neighbors of         N3 Check
```

Author Response: The dataset was collected at 295 K, therefore there is more vibrational motion of the atoms leading to larger displacement ellipsoids. This is especially the case for atoms with more vibrational/rotational freedom (such as N1, C1) or for atoms in disordered molecules (N2, N3). However, modeling these atoms as disordered did not improve the model

```
PLAT242_ALERT_2_C Low    'MainMol' Ueq as Compared to Neighbors of        Pb1 Check
```

Author Response: Atoms that have more restricted vibrational motion due to their coordination/bonding will likely have smaller displacement ellipsoids than their neighboring atoms (for example, Pb1 or C2). Modeling the surrounding atoms as disordered did not improve the refinement.

```
PLAT242_ALERT_2_C Low    'MainMol' Ueq as Compared to Neighbors of         C2 Check
```

Author Response: Atoms that have more restricted vibrational motion due to their coordination/bonding will likely have smaller displacement ellipsoids than their neighboring atoms (for example, Pb1 or C2). Modeling the surrounding atoms as disordered did not improve the refinement.

```
PLAT260_ALERT_2_C Large Average Ueq of Residue Including        N1      0.114 Check
```

Author Response: The dataset was collected at 295 K, therefore there is more vibrational motion of the atoms leading to larger displacement ellipsoids. This is especially the case for atoms with more vibrational/rotational freedom (such as N1, C1) or for atoms in disordered molecules (N2, N3). However, modeling these atoms as disordered did not improve the model.

```
PLAT260_ALERT_2_C Large Average Ueq of Residue Including        N2      0.102 Check
```

Author Response: The dataset was collected at 295 K, therefore there is more vibrational motion of the atoms leading to larger displacement ellipsoids. This is especially the case for atoms with more vibrational/rotational freedom (such as N1, C1) or for atoms in disordered molecules (N2, N3). However, modeling these atoms as disordered did not improve the model.

```
PLAT360_ALERT_2_C Short  C(sp3)-C(sp3) Bond  C5       - C6       .       1.40 Ang.
```

Author Response: The dataset was collected at room temperature yielding larger displacement ellipsoids due to thermal motion. Therefore, it is not unusual that the bond distances are slightly shorter than typical.

```
PLAT906_ALERT_3_C Large K Value in the Analysis of Variance ......      5.322 Check
```

Author Response: Usually data integration algorithms underestimate the background and prodcue slightly elevated values of Fo, resulting in higher Fo/Fc values. These are most pronounced for the weakest intensities.

```
PLAT911_ALERT_3_C Missing FCF Refl Between Thmin & STh/L=    0.600          8 Report
```

Author Response: All 8 of these reflections were collected. Three of these reflections were excluded during data integration and the remaining five were excluded during scaling.

Alert level G

```
PLAT002_ALERT_2_G Number of Distance or Angle Restraints on AtSite         10 Note
PLAT003_ALERT_2_G Number of Uiso or Uij Restrained non-H Atoms ...          4 Report
PLAT004_ALERT_5_G Polymeric Structure Found with Maximum Dimension          2 Info
PLAT007_ALERT_5_G Number of Unrefined Donor-H Atoms .............          11 Report
PLAT176_ALERT_4_G The CIF-Embedded .res File Contains SADI Records          2 Report
PLAT177_ALERT_4_G The CIF-Embedded .res File Contains DELU Records          1 Report
PLAT178_ALERT_4_G The CIF-Embedded .res File Contains SIMU Records          1 Report
PLAT187_ALERT_4_G The CIF-Embedded .res File Contains RIGU Records          1 Report
PLAT190_ALERT_3_G A Non-default RIGU Restraint Value for First Par     0.0200 Report
PLAT190_ALERT_3_G A Non-default RIGU Restraint Value for SecondPar     0.0200 Report
PLAT232_ALERT_2_G Hirshfeld Test Diff (M-X)  Pb1      --I1       .       11.4 s.u.
PLAT232_ALERT_2_G Hirshfeld Test Diff (M-X)  Pb1      --I2       .        6.5 s.u.
PLAT232_ALERT_2_G Hirshfeld Test Diff (M-X)  Pb1      --I3       .        7.8 s.u.
PLAT232_ALERT_2_G Hirshfeld Test Diff (M-X)  Pb1      --I3_d     .        9.3 s.u.
PLAT232_ALERT_2_G Hirshfeld Test Diff (M-X)  Pb1      --I1_e     .       13.2 s.u.
PLAT302_ALERT_4_G Anion/Solvent/Minor-Residue Disorder (Resd  3  )        33% Note
PLAT720_ALERT_4_G Number of Unusual/Non-Standard Labels .........           8 Note
PLAT794_ALERT_5_G Tentative Bond Valency for Pb1       (II)      .       2.27 Info
PLAT860_ALERT_3_G Number of Least-Squares Restraints ............          90 Note
PLAT870_ALERT_4_G ALERTS Related to Twinning Effects Suppressed ..          ! Info
PLAT912_ALERT_4_G Missing # of FCF Reflections Above STh/L=  0.600         58 Note
PLAT951_ALERT_5_G Calculated (ThMax) and CIF-Reported Kmax Differ           2 Units
PLAT957_ALERT_1_G Calculated (ThMax) and Actual (FCF) Kmax Differ           2 Units
PLAT992_ALERT_5_G Repd & Actual _reflns_number_gt Values Differ by          2 Check
```

---

0 **ALERT level A** = Most likely a serious problem - resolve or explain
1 **ALERT level B** = A potentially serious problem, consider carefully
12 **ALERT level C** = Check. Ensure it is not caused by an omission or oversight
24 **ALERT level G** = General information/check it is not something unexpected

2 ALERT type 1 CIF construction/syntax error, inconsistent or missing data
16 ALERT type 2 Indicator that the structure model may be wrong or deficient
6 ALERT type 3 Indicator that the structure quality may be low
8 ALERT type 4 Improvement, methodology, query or suggestion
5 ALERT type 5 Informative message, check

It is advisable to attempt to resolve as many as possible of the alerts in all categories. Often the minor alerts point to easily fixed oversights, errors and omissions in your CIF or refinement strategy, so attention to these fine details can be worthwhile. In order to resolve some of the more serious problems it may be necessary to carry out additional measurements or structure refinements. However, the purpose of your study may justify the reported deviations and the more serious of these should normally be commented upon in the discussion or experimental section of a paper or in the "special_details" fields of the CIF. checkCIF was carefully designed to identify outliers and unusual parameters, but every test has its limitations and alerts that are not important in a particular case may appear. Conversely, the absence of alerts does not guarantee there are no aspects of the results needing attention. It is up to the individual to critically assess their own results and, if necessary, seek expert advice.

Publication of your CIF in IUCr journals

A basic structural check has been run on your CIF. These basic checks will be run on all CIFs submitted for publication in IUCr journals (*Acta Crystallographica*, *Journal of Applied Crystallography*, *Journal of Synchrotron Radiation*); however, if you intend to submit to *Acta Crystallographica Section C* or *E* or *IUCrData*, you should make sure that full publication checks are run on the final version of your CIF prior to submission.

Publication of your CIF in other journals

Please refer to the *Notes for Authors* of the relevant journal for any special instructions relating to CIF submission.

PLATON version of 28/11/2022; check.def file version of 28/11/2022

Datablock jin165_d - ellipsoid plot

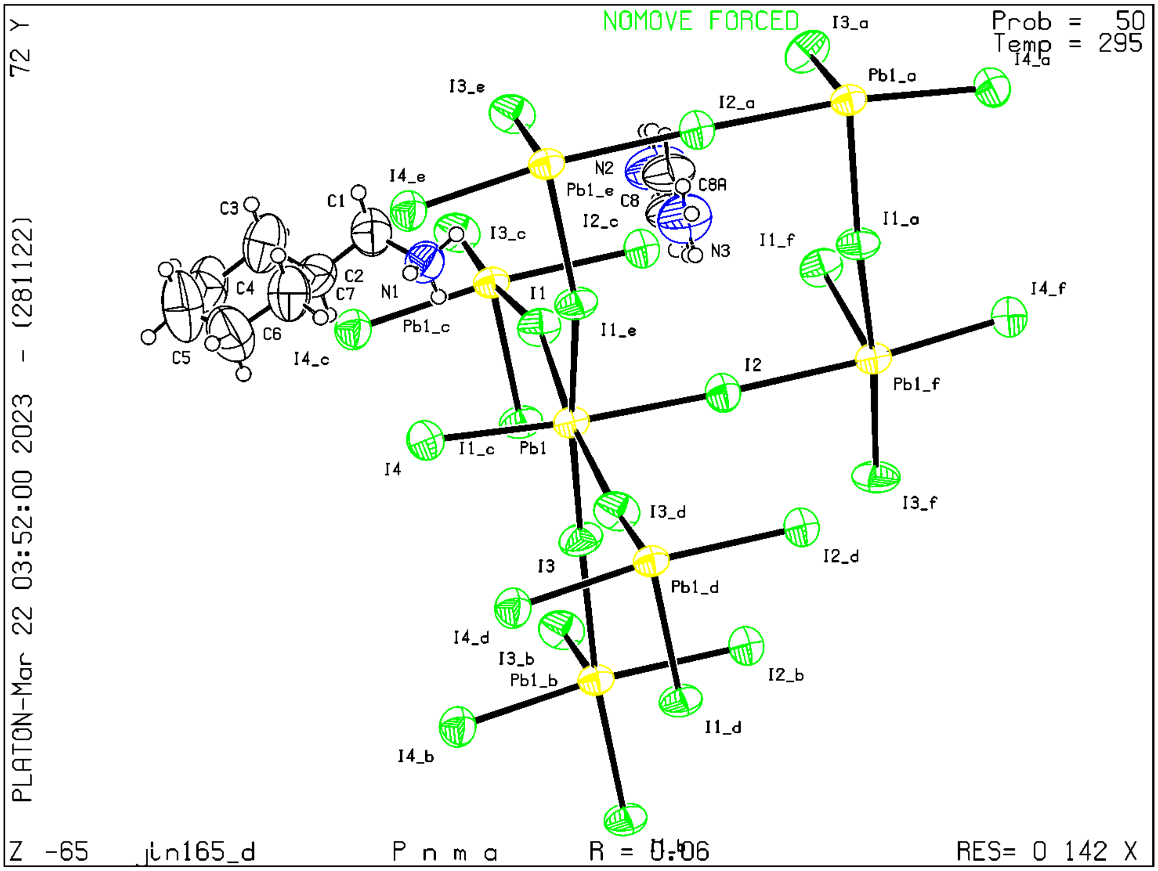

Supplement: Supplementary file 1 — Supplementary Information [file 41467_2024_47225_MOESM1_ESM.pdf]
